# Hölder inequality applied on a non-Newtonian fluid equation with a nonlinear convection term and a source term

**DOI:** 10.1186/s13660-018-1938-x

**Published:** 2018-12-13

**Authors:** Huashui Zhan

**Affiliations:** School of Applied Mathematics, Xiamen University of Technology, Xiamen, China

**Keywords:** 35K65, 35K57, 35R35, Non-Newtonian fluid equation, The Hölder inequality, Nonlinear convection term, The optimal partial boundary value condition

## Abstract

Consider a non-Newtonian fluid equation with a nonlinear convection term and a source term. The existence of the weak solution is proved by Simon’s compactness theorem. By the Hölder inequality, if both the diffusion coefficient and the convection term are degenerate on the boundary, then the stability of the weak solutions may be proved without the boundary value condition. If the diffusion coefficient is only degenerate on a part of the boundary value, then a partial boundary value condition is required. Based on this partial boundary, the stability of the weak solutions is proved. Moreover, the uniqueness of the weak solution is proved based on the optimal boundary value condition.

## Introduction and the main results

The evolutionary equation related to the *p*-Laplacian

1.1 $$ {u_{t}} = \operatorname{div} \bigl(a(x){ \vert {\nabla u} \vert ^{p - 2}}\nabla u \bigr) $$

arises in the fields of mechanics, physics and biology. For instance, in the theory of non-Newtonian fluids, the quantity *p* is a characteristic of the medium, the media with $p > 2$ are called dilatant fluids and those with $p < 2$ are called pseudoplastics; if $p=2$ they are Newtonian fluids. If $a(x)\equiv1$, there is a tremendous amount of work on the existence, the uniqueness and the regularity of the weak solutions of the equation, one can refer to Refs. [1–7] and the references therein. Zhao [8] had studied the equation

1.2 $$ {u_{t}} = \operatorname{div} \bigl({ \vert {\nabla u} \vert ^{p - 2}}\nabla u \bigr) +f(\nabla u, u,x,t), $$

and revealed some essential differences coming from the term $f(\nabla u, u,x,t)$. Yin–Wang [9] had studied the equation

1.3 $$ \frac{\partial u}{\partial t}-\operatorname{div} \bigl(a(x)|\nabla u|^{p-2}\nabla u \bigr)-b_{i}(x)D_{i}u+c(x,t)u=f(x,t), $$

revealed how the degeneracy of the diffusion coefficient $a(x)$ affects the boundary value condition, where $D_{i} =\frac{\partial}{\partial x_{i}}$, $a \in C(\overline{\varOmega})$ and $a(x)\geq0$.

In this paper, we consider

1.4 $$ {u_{t}} = \operatorname{div} \bigl({a(x)} { \vert {\nabla u} \vert ^{p - 2}}\nabla u \bigr) +\sum_{i = 1}^{N} \frac{\partial b_{i}(u,x,t)}{\partial x_{i}}+f(u,x,t) , \quad (x,t) \in{Q_{T}}, $$

where *Ω* is a bounded domain in $\mathbb{R}^{N}$ with appropriately smooth boundary, $p>1$, ${Q_{T}} = \varOmega \times(0,T)$, $a(x) \in C^{1}(\overline{\varOmega})$, $a(x)\geq0$ and

1.5 $$ a(x)> 0,\quad x\in\varOmega, $$

the nonlinear convection $b_{i}(s,x,t)\in C(\mathbb{R}\times\overline {Q_{T}})$, the source term $f(s,x,t)\in C(\mathbb{R}\times\overline {Q_{T}})$. Comparing with [9], we must pay attention on how these two nonlinear terms affect the well-posedness problem of Eq. (1.4).

The condition (1.5) guarantees that Eq. (1.4) has not hyperbolic character. In other words, if the set $\{x\in\varOmega: a(x)=0\}$ has an interior point, then Eq. (1.4) is with a hyperbolic-parabolic type, the uniqueness of the solution may be obtained only in the sense of the entropy solution. Since the condition (1.5), $a(x)$ only may be degenerate on the boundary, Eq. (1.4) is a sheer degenerate parabolic equation. Thus, we can discuss its well-posedness of the usual weak solutions instead of the entropy solution.

Drawing on the experience of the linear degenerate parabolic theory, to study the well-posedness of the solutions of Eq. (1.4), the initial value

1.6 $$ u(x,0)=u_{0}(x),\quad x\in\varOmega, $$

is always necessary. While, the usual Dirichlet boundary value condition

1.7 $$ u(x,t)=0, \quad (x,t)\in\partial\varOmega\times(0,T), $$

may be overdetermined. So it is only a partial boundary condition

1.8 $$ u(x,t)=0, \quad (x,t)\in\varSigma_{p}\times(0,T), $$

imposed in [9], where $\varSigma_{p}\subseteq\partial\varOmega$. In particular, if $\varSigma_{p}=\varnothing$, then there is not any boundary value condition. But the partial boundary condition [9] is in a weaker sense than the trace.

The methods used in what follows are different from those in [9], we still use the sense of the trace to define the boundary value condition (1.7) or (1.8). Roughly speaking, we will show that the condition

1.9 $$ a(x)=b_{i}(\cdot, x, t)=0,\quad x\in\partial\varOmega, $$

can substitute the boundary value condition (1.7). But if (1.9) is not right, only the partial boundary value condition (1.8) is required, we need to find the explicit formulas of $\varSigma_{p}$ and judge which one is the best.

The definition of the weak solutions follows a Banach space which is defined as follows. For every fixed $t\in[0, T]$, let

1.10 $$ \begin{aligned} &V_{t}(\varOmega) = \bigl\{ u(x) : u(x)\in L^{2}(\varOmega)\cap W^{1,1}_{0}(\varOmega), \bigl|\nabla u(x)\bigr|^{p}\in L^{1} (\varOmega) \bigr\} , \\ &\|u\|_{V_{t}(\varOmega)} = \|u\|_{2,\varOmega} + \|\nabla u\|_{p,\varOmega} , \end{aligned} $$

and denote by $V'_{t}(\varOmega)$ its dual space. By $\mathbf{W}(Q_{T})$ we denote the Banach space

1.11 $$ \textstyle\begin{cases} \mathbf{W}(Q_{T}) = \{u : [0,T]\rightarrow V_{t}(\varOmega)|u\in L^{2}(Q_{T}),|\nabla u|^{p} \in L^{1}(Q_{T}), u = 0\mbox{ on }\varGamma \}, \\ \|u\|_{\mathbf{W}(Q_{T})} = \|\nabla u\|_{p,Q_{T}} + \|u\|_{2,Q_{T}} . \end{cases} $$

$\mathbf{W}'(Q_{T})$ is the dual space of $\mathbf{W}(Q_{T})$ (the space of linear functionals over $\mathbf{W}(Q_{T})$).

### Definition 1.1

A function $u(x,t)$ is said to be a weak solution of Eq. (1.4) with the initial value (1.6), if

1.12 $$ u \in{L^{\infty}}({Q_{T}}),\qquad {a(x)} { \vert {\nabla u} \vert ^{p}} \in {L^{1}}({Q_{T}}),\qquad u_{t}\in\mathbf{W}'(Q_{T}) $$

and for any function $\varphi_{1} \in L^{\infty}(0,T; W_{0}^{1,p}(\varOmega ))$, $\varphi_{2}\in L^{1}(0,T; W^{1,p}_{\mathrm{loc}}(\varOmega))$,

1.13 $$\begin{aligned}& \langle\!\langle u_{t},\varphi_{1}\varphi_{2} \rangle\!\rangle+ \iint_{Q_{T}}a(x) \vert \nabla u \vert ^{p - 2}\nabla u \cdot\nabla(\varphi_{1}\varphi_{2}) + \sum _{i = 1}^{N} b_{i}(u,x,t) \cdot( \varphi_{1}\varphi_{2})_{x_{i}}\,dx\,dt \\& \quad = \iint_{Q_{T}}f(u,x,t)\varphi_{1}\varphi_{2} \,dx\,dt. \end{aligned}$$

The initial value is satisfied in the sense of that

1.14 $$ \lim_{t\rightarrow0} \int_{\varOmega} \bigl\vert u(x,t)-u_{0}(x) \bigr\vert \,dx=0. $$

### Definition 1.2

Let $p>1$. The function $u(x,t)$ is said to be the weak solution of Eq. (1.4) with the initial boundary values (1.6)–(1.7) (or (1.8)) if *u* satisfies Definition 1.1, and the boundary condition (1.7) (or (1.8) respectively) is satisfied in the sense of trace.

### Theorem 1.3

*If* $p>1$, $b_{i}(s,x,t)$ *and* $f(s,x,t)$ *are* $C^{1}(\mathbb{R}\times\overline{\varOmega}\times[0,T])$ *functions*, *and*

1.15 $$ {u_{0}} \in{L^{\infty}}(\varOmega), \qquad {a(x) } { \vert { \nabla{u_{0}}} \vert ^{p}} \in{L^{1}}( \varOmega), $$

*then Eq*. (1.4) *with initial value* (1.6) *has a weak solution*.

### Theorem 1.4

*Let* $p>1$, $\int_{\varOmega}a(x)^{-\frac {1}{p-1}}\,dx<\infty$, $b_{i}(s, x, t)$ *and* $f(s,x,t)$ *be* $C(\mathbb {R}\times\overline{\varOmega}\times[0,T])$ *functions*. *Then the initial boundary value problem* (1.4)*–*(1.6) *and* (1.7) (*or* (1.8)) *has a solution*.

The first aim of this paper is to prove the following stability theorems without any boundary value condition.

### Theorem 1.5

*Let* $u(x,t)$, $v(x,t)$ *be two solutions of* (1.4) *with the initial values* $u_{0}(x)$, $v_{0}(x)$, *respectively*. *If there is a function* $g_{i}(x)$ *with* $g_{i}(x)|_{x\in\partial\varOmega}=0$ *such that*

1.16 $$ \bigl\vert b_{i}(u,x,t)-b_{i}(v,x,t) \bigr\vert \leq c g_{i}(x) \vert u-v \vert ,\qquad \int_{\varOmega } \bigl\vert g_{i}(x) \bigr\vert ^{\frac{p}{p-1}}a(x)^{-\frac{1}{p-1}}\,dx< \infty, $$

$f(s,x,t)$ *is a Lipschitz function and* $a(x)$ *satisfies*

1.17 $$ a(x)|_{x\in\partial\varOmega}=0, \qquad n \int_{\varOmega\setminus\varOmega _{\frac{1}{n}}}a(x)|\nabla a|^{p}\,dx\leq c, $$

*then*

1.18 $$ \int_{\varOmega} \bigl\vert u(x,t)-v(x,t) \bigr\vert \,dx\leq \int_{\varOmega} \bigl\vert u_{0}(x)-v_{0}(x) \bigr\vert \,dx. $$

*The stability* (1.17) *is true*.

Here and the hereafter, for any positive small $\delta>0$, $\varOmega _{\delta}=\{x\in\varOmega: a(x)>\delta\}$.

An interesting corollary from Theorem 1.5 is that, if $\int_{\varOmega }a(x)^{-\frac{1}{p-1}}\,dx<\infty$, then without the condition (1.16), only if the condition (1.17) holds, the stability (1.18) is true. Additionally, the second inequality of (1.16) implies that $g_{i}(x)|_{x\in\partial\varOmega}=0$. In fact, the condition (1.17) can be replaced by the other conditions. The following theorem is one of results expected.

### Theorem 1.6

*Let* $u(x,t)$, $v(x,t)$ *be two solutions of* (1.4) *with the initial values* $u_{0}(x)$, $v_{0}(x)$, *respectively*. *If there is a function* $g_{i}(x)$ *with* $g_{i}(x)|_{x\in\partial\varOmega}=0$ *such that* (1.16) *is true* $f(s,x,t)$ *is a Lipschitz function and* $a(x)$ *satisfies*

1.19 $$ a(x)|_{x\in\partial\varOmega}=0, \qquad \int_{\varOmega}a(x)^{1-p}|\nabla a|^{p}\,dx< \infty, $$

*then the stability* (1.18) *is true*.

Moreover, by choosing suitable test function, using the Hölder inequality, we can prove another stability theorem without any boundary value condition.

### Theorem 1.7

*Let* $u(x,t)$, $v(x,t)$ *be two solutions of* (1.4) *with the initial values* $u_{0}(x)$, $v_{0}(x)$, *respectively*. *If* $f(s,x,t)$ *and* $b_{i}(s,x,t)$ *are Lipschitz functions*, $a(x)$ *satisfies*

1.20 $$ a(x)|_{x\in\partial\varOmega}=0, \qquad \int_{\varOmega_{\frac {1}{m}}\setminus \varOmega_{\frac{2}{m}}}\frac{|\nabla a|}{a(x)-\frac {1}{m}}\,dx< \infty, $$

*then the stability* (1.18) *is true*.

The second aim of this paper is to prove the stability theorems based on the partial boundary value condition (1.8).

### Theorem 1.8

*Let* $u(x,t)$, $v(x,t)$ *be two solutions of* (1.4) *with the initial values* $u_{0}(x)$, $v_{0}(x)$, *respectively*, *and with the same partial boundary value condition*

1.21 $$ u(x,t)=v(x,t)=0, \quad (x,t)\in\varSigma_{p}\times(0,T), $$

*where*

1.22 $$ \varSigma_{p}=\varSigma_{1}=\bigl\{ x\in\partial\varOmega: a(x)a_{x_{i}}\neq0\bigr\} . $$

*If* $a(x)$ *satisfies* (1.5) *and*

1.23 $$ n \int_{\varOmega\setminus\varOmega_{\frac{1}{n}}}a(x)|\nabla a|^{p}\,dx\leq c, \qquad \int_{\varOmega}a(x)^{-\frac{1}{p-1}}\,dx< \infty, $$

$f(s,x,t)$ *and* $b_{i}(s,x,t)$ *are Lipschitz functions*, *then the stability* (1.18) *is true*.

We emphasize that the conditions (1.16), (1.17), (1.19), (1.20) and (1.23) are used to prove the stability of the weak solutions. In fact, only if $a(x)$ satisfies (1.5), the uniqueness is always true.

### Theorem 1.9

*Let* $p>1$, $b_{i}(s, x, t)$ *be a Lipschitz function*, $f(s,x,t)$ *is a continuous function*. *If* $u(x,t)$, $v(x,t)$ *are two solutions of Eq*. (1.4) *with the initial values* $u_{0}(x)$, $v_{0}(x)$, *respectively*, *with the same partial boundary value condition* (1.21) *in which*

1.24 $$ \varSigma_{p}=\varSigma_{2}=\bigl\{ x\in\partial\varOmega: a(x)\neq0 \bigr\} , $$

*then there exists a positive constant* $\beta\geq2$ *such that*

1.25 $$ \int_{\varOmega}a^{\beta} \bigl\vert u(x,t)-v(x,t) \bigr\vert ^{2}\,dx\leq c \int_{\varOmega }a^{\beta} \bigl\vert u_{0}(x)-v_{0}(x) \bigr\vert ^{2}\,dx. $$

*In particular*, *for any small enough constant* $\delta>0$,

1.26 $$ \int_{\varOmega_{\delta}} \bigl\vert u(x,t)-v(x,t) \bigr\vert ^{2}\,dx\leq c\delta^{-\beta } \int_{\varOmega} \bigl\vert u_{0}(x)-v_{0}(x) \bigr\vert ^{2}\,dx, $$

*where* $\varOmega_{\delta}=\{x\in\varOmega: a(x)>\delta\}$ *as before*.

From this theorem, if $u_{0}(x)=v_{0}(x)$, by the arbitrariness of *δ* in (1.26), the solution of Eq. (1.4) with the initial value and the partial boundary value condition (1.24) is unique. We can see that, if $\varSigma_{2}=\partial\varOmega$, i.e., $a(x)\geq c>0$, Eq. (1.4) is similar to the classical evolutionary *p*-Laplacian equation, (1.24) is the usual Dirichlet boundary value condition, the uniqueness is true naturally. While $\varSigma_{2}=\emptyset$, i.e., $a(x)=0$ on the boundary *∂Ω*, the uniqueness of the weak solution is true independent of the boundary value condition. If $b_{i}(u,x,t)=b_{i}(u)$, $u_{t}\in L^{2}(Q_{T})$ and $a(x)=d^{\alpha}(x)$ where $d(x)=\operatorname {dist}(x, \partial\varOmega)$, the same conclusion as of Theorem 1.9 had been proved by the author in his previous work [10]. So, essential progress of this paper is that we do not assume that $a(x)|_{x\in \partial\varOmega}=0$, the best partial boundary value condition is (1.24). This fact also remains an open problem: whether the partial boundary value condition (1.21) can be replaced by (1.24).

## The existence of the weak solutions

This section considers the weak solution of the initial-value problem for Eq. (1.4). It is supposed that $u_{0}$ satisfies (1.15)

By the results of [10, Sect. 8], also referring to [11], we have the following important lemma.

### Lemma 2.1

*If* $u_{\varepsilon}\in L^{\infty}(0,T;L^{2}(\varOmega ))\cap\mathbf{W}(Q_{T})$, $\Vert u_{\varepsilon t} \Vert _{\mathbf{W}'(Q_{T})}\leq c$, $\|\nabla(|u_{\varepsilon }|^{q-1}u_{\varepsilon})\|_{p,Q_{T}}\leq c$, *then there is a subsequence of* $\{u_{\varepsilon}\}$ *which is relatively compactness in* $L^{s}(Q_{T})$ *with* $s\in(1,\infty)$. *Here* $q\geq1$.

We consider the following regularized problem:

2.1 $$\begin{aligned}& u_{\varepsilon t} - \operatorname{div} \bigl(\bigl(a(x)+\varepsilon\bigr) \bigl( \vert \nabla{u_{\varepsilon}} \vert ^{2}+\varepsilon\bigr)^{\frac{p - 2}{2}}\nabla{u_{\varepsilon}}) - \sum _{i = 1}^{N} \frac{\partial b_{i}(u_{\varepsilon},x,t)}{\partial x_{i}} \\& \quad =f(u_{\varepsilon}, x,t),\quad (x,t)\in {Q_{T}}, \end{aligned}$$

2.2 $$\begin{aligned}& {u_{\varepsilon}}(x,t) = 0,\quad (x,t) \in\partial\varOmega \times (0,T), \end{aligned}$$

2.3 $$\begin{aligned}& {u_{\varepsilon}}(x,0) = {u_{\varepsilon,0}}(x), \quad x\in\varOmega . \end{aligned}$$

For any ${u_{\varepsilon,0}} \in{C^{\infty}_{0} }(\varOmega)$, ${a(x) \vert {\nabla{u_{\varepsilon,0}}} \vert ^{p}}$ uniformly is convergent to $a(x)|\nabla u_{0}(x)|^{p}$ in ${L^{1}}(\varOmega)$, it is well known that the above problem has a unique classical solution [12, 13].

According to the maximum principle [2], there is a constant *c* only dependent on $\Vert {{u_{0}}} \Vert _{{L^{\infty}}(\varOmega)}$ but independent on *ε*, such that

2.4 $$ \Vert {{u_{\varepsilon}}} \Vert _{{L^{\infty}}({Q_{T}})} \leqslant c. $$

Multiplying (2.1) by $u_{\varepsilon}$ and integrating it over $Q_{T}$, we easily have

2.5 $$\begin{aligned}& \frac{1}{2} \int_{\varOmega}{u_{\varepsilon}^{2}\, {d}x} + \iint _{{Q_{T}}} \bigl(a(x)+\varepsilon\bigr){\bigl( \vert \nabla u_{\varepsilon} \vert ^{2}+\varepsilon \bigr)^{\frac{p-2}{2}} \vert \nabla u_{\varepsilon} \vert ^{2}\, {d}x\, {d}t} \\& \quad \leq \iint_{{Q_{T}}} \bigl\vert f(u_{\varepsilon},x,t)u_{\varepsilon}\bigr\vert \leqslant c. \end{aligned}$$

For small enough $\delta>0$, since $p>1$, by (1.5) and (2.5),

2.6 $$ \int_{0}^{T} \int_{\varOmega_{\delta}}|\nabla u_{\varepsilon}|\,dx\,dt\leq c \biggl( \int_{0}^{T} \int_{\varOmega_{\delta}}|\nabla u_{\varepsilon }|^{p}\,dx\,dt \biggr)^{\frac{1}{p}}\leq c(\delta). $$

Also

2.7 $$ \iint_{{Q_{T}}} a(x)|\nabla u_{\varepsilon}|^{p}\,dx \,dt\leqslant c \iint _{{Q_{T}}}\bigl(a(x)+\varepsilon\bigr)|\nabla u_{\varepsilon}|^{p}\,dx\,dt\leqslant c. $$

Now, for any $v\in\mathbf{W}(Q_{T})$, $\|v\|_{W(Q_{T})}=1$,

$$\begin{aligned} \langle u_{\varepsilon t}, v\rangle =&- \iint_{Q_{T}}\bigl(a(x)+\varepsilon\bigr) \bigl(|\nabla u_{\varepsilon}|^{2} +\varepsilon \bigr)^{\frac{p-2}{2}}\nabla u_{\varepsilon}\cdot\nabla v \,dx\,dt \\ &{}- \iint_{Q_{T}}\frac{\partial v}{\partial x_{i}}{b_{i}}({u_{\varepsilon}},x,t) \,dx\,dt + \iint_{Q_{T}}f(u_{\varepsilon},x,t)v\,dx\,dt. \end{aligned}$$

Using the Young inequality, we can show that

$$\bigl\vert \langle u_{\varepsilon t}, v\rangle \bigr\vert \leq c \biggl[1+ \iint_{{Q_{T}}} \bigl(a(x)+\varepsilon\bigr)|\nabla u_{\varepsilon}|^{p}\,dx\,dt+ \iint_{{Q_{T}}} \bigl(|v|^{p}+|\nabla v|^{p} \bigr)\,dx\,dt \biggr]\leq c, $$

then

2.8 $$ \| u_{\varepsilon t}\|_{\mathbf{W}'(Q_{T})}\leq c. $$

Now, let $\varphi\in C_{0}^{1}(\varOmega)$, $0\leq\varphi\leq1$ such that

$$\varphi|_{\varOmega_{2\delta}}=1, \qquad \varphi|_{\varOmega\setminus \varOmega_{\delta}}=0. $$

Then

$$\bigl\vert \bigl\langle (\varphi u_{\varepsilon})_{t}, v\bigr\rangle \bigr\vert = \bigl\vert \langle \varphi u_{\varepsilon t}, v\rangle \bigr\vert \leq \bigl\vert \langle u_{\varepsilon t}, v\rangle \bigr\vert , $$

we have

2.9 $$\begin{aligned}& \bigl\Vert \bigl(\varphi(x) u\bigr)_{\varepsilon t} \bigr\Vert _{\mathbf{W}'(Q_{T})}\leq \Vert u_{\varepsilon t} \Vert _{\mathbf{W}'(Q_{T})}\leq c, \end{aligned}$$

2.10 $$\begin{aligned}& \iint_{Q_{T}} \bigl\vert \nabla(\varphi u_{\varepsilon}) \bigr\vert ^{p}\,dx\,dt\leq c(\delta ) \biggl(1+ \int_{0}^{T} \int_{\varOmega_{\delta}} \vert \nabla u_{\varepsilon} \vert ^{p} \,dx\,dt\biggr)\leq c(\delta), \end{aligned}$$

and so

2.11 $$ \bigl\Vert \nabla(\varphi u_{\varepsilon}) \bigr\Vert _{p,Q_{T}}\leq c. $$

By Lemma 2.1, $\varphi u_{\varepsilon}$ is relatively compact in $L^{s}(Q_{T})$ with $s\in(1,\infty)$. Then $\varphi u_{\varepsilon}\rightarrow\varphi u$ a.e. in $Q_{T}$. In particular, due to the arbitrariness of *δ*, $u_{\varepsilon}\rightarrow u$ a.e. in $Q_{T}$.

Hence, by (2.4), (2.7), there exists a function *u* and an *n*-dimensional vector function $\overrightarrow{\zeta}= ({\zeta_{1}}, \ldots,{\zeta_{n}})$ satisfying

$$u \in L^{\infty}(Q_{T}),\qquad \vert {\overrightarrow{\zeta}} \vert \in{L^{\frac{p}{{p - 1}}}}({Q_{T}}), $$

and

$$\begin{aligned}& {u_{\varepsilon}} \rightharpoonup * u,\quad \text{in } {L^{\infty}(Q_{T})}, \qquad u_{\varepsilon}\rightarrow u,\quad \text{a.e. in } Q_{T}, \\& b_{i}(u_{\varepsilon},x,t)\rightarrow b_{i}(u,x,t),\quad \text{a.e. in } Q_{T}, \\& f(u_{\varepsilon},x,t)\rightarrow f(u,x,t),\quad \text{a.e. in } Q_{T}, \\& {\nabla u_{\varepsilon}} \rightharpoonup\nabla u \quad \text{in } \bigl(L_{\mathrm{loc}}^{p}(Q_{T}) \bigr)^{N}, \\& \bigl(a(x)+\varepsilon\bigr) { \vert {\nabla{u_{\varepsilon}}} \vert ^{p - 2}}\nabla{u_{\varepsilon}} \rightharpoonup\overrightarrow{\zeta}\quad \text{in } \bigl({L^{\frac{p}{p-1}}}({Q_{T}}) \bigr)^{N}. \end{aligned}$$

Similar to the evolutionary *p*-Laplacian equation, we can prove that

2.12 $$ \iint_{Q_{T}} a(x) \vert \nabla u \vert ^{p - 2}\nabla u \cdot\nabla \varphi \, dx\, dt = \iint_{Q_{T}} \overrightarrow{\zeta}\cdot\nabla\varphi\, {d}x\, dt, $$

for any function $\varphi \in C_{0}^{1} ({Q_{T}})$. Then

2.13 $$\begin{aligned}& \langle\!\langle u_{t},\varphi\rangle\!\rangle+ \iint_{{Q_{T}}} \Biggl[a(x) \vert \nabla u \vert ^{p- 2} \nabla u \cdot\nabla\varphi+\sum_{i=1}^{N}b_{i}(u,x,t) \varphi_{x_{i}} \Biggr]\,dx\,dt \\& \quad = \iint_{Q_{T}}f(u,x,t)\varphi \,dx\,dt. \end{aligned}$$

If we denote $\varOmega_{\varphi}=\operatorname{supp}\varphi$, then

2.14 $$\begin{aligned}& \langle\!\langle u_{t},\varphi\rangle\!\rangle+ \int_{0}^{T} \int_{\varOmega_{\varphi}} \Biggl[a(x) \vert \nabla u \vert ^{p- 2} \nabla u \cdot\nabla\varphi+\sum_{i=1}^{N}b_{i}(u,x,t) \varphi_{x_{i}} \Biggr]\,dx\,dt \\& \quad = \int_{0}^{T} \int_{\varOmega_{\varphi}}f(u,x,t)\varphi \,dx\,dt. \end{aligned}$$

Now, for any $\varphi_{1}\in C_{0}^{1} ({Q_{T}})$, $\varphi_{2}(x,t)\in L^{1}(0,T; W^{1,p}_{\mathrm{loc}}(\varOmega))$, it is clearly that

$$\varphi_{2}\in L^{1}(0,T; W^{1,p}( \varOmega_{\varphi_{1}}). $$

By the fact that $C_{0}^{\infty}(\varOmega_{\varphi_{1}})$ is dense in $W^{1, p}(\varOmega_{\varphi_{1}})$, by a limit process, we have

2.15 $$\begin{aligned}& \langle\!\langle u_{t},\varphi_{1}\varphi_{2} \rangle\!\rangle+ \int_{0}^{T} \int_{\varOmega_{\varphi _{1}}} \bigl[a(x) \vert \nabla u \vert ^{p- 2} \nabla u \cdot\nabla (\varphi_{1}\varphi_{2}) +b_{i}(u,x,t) (\varphi_{1}\varphi_{2})_{x_{i}} \bigr]\,dx\,dt \\& \quad = \int_{0}^{T} \int_{\varOmega_{\varphi_{1}}}f(u,x,t) (\varphi_{1}\varphi_{2}) \,dx\,dt, \end{aligned}$$

which implies that

2.16 $$\begin{aligned}& \langle\!\langle u_{t},\varphi_{1}\varphi_{2} \rangle\!\rangle+ \int_{0}^{T} \int_{\varOmega} \bigl[ a(x) \vert \nabla u \vert ^{p- 2} \nabla u \cdot\nabla(\varphi _{1}\varphi_{2})+b_{i}(u,x,t) (\varphi_{1}\varphi_{2})_{x_{i}} \bigr]\,dx\,dt \\& \quad = \int_{0}^{T} \int_{\varOmega}f(u,x,t) (\varphi_{1}\varphi_{2}) \,dx\,dt. \end{aligned}$$

Again by a limit process, $\varphi_{1}$ can be chosen as in Definition 1.1.

At last, we are able to prove (1.14) as in [14], then *u* is a solution of Eq. (1.4) with the initial value (1.6) in the sense of Definition 1.1. Thus we have Theorem 1.3. By a similar method to [15], one easily proves the following lemma, we omit the details here.

### Lemma 2.2

*If* $\int_{\varOmega}a(x)^{-\frac{1}{p-1}}\,dx<\infty $, *u* *is a weak solution of Eq*. (1.4) *with the initial value* (1.6). *Then*, *for any given* $t\in[0,T)$, $u\in W^{1,\gamma}(\varOmega)$ *for some* $\gamma>1$, *and the trace of* *u* *on the boundary* *∂Ω* *can be defined in the traditional way*.

By Theorem 1.3 and Lemma 2.2, we have Theorem 1.4 clearly.

## The stability without the boundary value condition

For any given positive integer *n*, let ${g_{n}}(s)$ be an odd function, and

$${g_{n}}(s) = \textstyle\begin{cases} 1,& s > \frac{1}{n}, \\ {n^{2}}{s^{2}}{{\mathrm{e}}^{1 - {n^{2}}{s^{2}}}},& 0\leq s \leqslant\frac{1}{n}. \end{cases} $$

Clearly, if denoting $G_{n}(s)=\int_{0}^{s}g_{n}(s)\,ds$, then

$$\lim_{n\rightarrow0}g_{n}(s)=\operatorname{sgn}(s), \qquad \lim_{n\rightarrow 0}G_{n}(s)=|s|, \quad s\in(-\infty, + \infty), $$

and by

$$g_{n}'(s) = \textstyle\begin{cases} 0,&s > \frac{1}{n}, \\ 2n^{2}se^{1-n^{2}s^{2}}(1-n^{2}s^{2}e^{1 - n^{2}s^{2}}), &0\leq s \leqslant\frac{1}{n}, \end{cases} $$

we have

$$\lim_{n\rightarrow0} sg_{n}'(s)=0, $$

where *c* is independent of *n*.

### Lemma 3.1

*Let* $u\in\mathbf{W}(Q_{T})$, $u_{t}\in\mathbf {W}'(Q_{T})$. *Then* ∀ *a*.*e*. $t_{1}, t_{2}\in(0, T)$,

$$\int_{t_{1}}^{t_{2}} \int_{\varOmega}uu_{t}\,dx\,dt=\frac{1}{2} \biggl[ \int _{\varOmega}\bigl(u^{2}(x,t_{2})-u^{2}(x,t_{1}) \bigr)\,dx \biggr]. $$

This is Corollary 2.1 of [11].

By a similar analysis, one can generalize Lemma 3.1.

### Lemma 3.2

*Let* $u\in\mathbf{W}(Q_{T})$, $u_{t}\in\mathbf {W}'(Q_{T})$. *For any continuous function* $h(s)$, $H(s)=\int_{0}^{s}h(s)\,ds$, *a*.*e*. $t_{1}, t_{2}\in(0, T)$,

$$\int_{t_{1}}^{t_{2}} \int_{\varOmega}h(u)u_{t}\,dx\,dt= \int_{\varOmega }\bigl(H(u) (x,t_{2})-H(u) (x,t_{1})\bigr)\,dx. $$

### Proof of Theorem 1.5

Let $u(x,t)$ and $v(x,t)$ be two weak solutions of Eq. (1.4) with the initial values $u(x,0)$, $v(x,0)$, respectively. Let

3.1 $$ \phi_{n}(x)= \textstyle\begin{cases} 1, & \mbox{if } x\in\varOmega_{\frac{1}{n}}, \\ na(x), & \mbox{if } x\in\varOmega\setminus\varOmega_{\frac{1}{n}}. \end{cases} $$

By a limit process, we can choose $\chi_{[\tau,s]}\phi_{n}{g_{n}}(u - v)$ as the test function, where $\chi_{[\tau,s]}$ is the characteristic function of $[\tau, s]\subset(0, T)$, then

3.2 $$\begin{aligned}& \int_{\tau}^{s} \int_{{\varOmega}} \phi_{n}(x){g_{n}}(u - v) \frac {\partial(u - v)}{\partial t}\,dx\,dt \\& \qquad {}+ \int_{\tau}^{s} \int_{{\varOmega}} a(x) \bigl( \vert \nabla u \vert ^{p - 2} \nabla u - \vert \nabla v \vert ^{p- 2}\nabla v \bigr) \cdot\nabla(u - v)g'_{n}(u-v)\phi_{n}(x)\,dx\,dt \\& \qquad {}+ \int_{\tau}^{s} \int_{{\varOmega}} a(x) \bigl( \vert \nabla u \vert ^{p(x) - 2} \nabla u - \vert \nabla v \vert ^{p(x) - 2}\nabla v \bigr) \cdot(u - v)g_{n}(u-v)\nabla\phi_{n}\, dx\, dt \\& \qquad {}+ \int_{\tau}^{s} \int_{{\varOmega}}\bigl[b_{i}(u,x,t)-b_{i}(v,x,t) \bigr]\phi _{nx_{i}}{g_{n}}(u - v)\,dx\,dt \\& \qquad {}+ \int_{\tau}^{s} \int_{{\varOmega }}\bigl[b_{i}(u,x,t)-b_{i}(v,x,t) \bigr](u-v)_{x_{i}}\phi_{n}{g'_{n}}(u - v)\,dx\,dt \\& \quad = \int_{\tau}^{s} \int_{{\varOmega}}\bigl[f(u,x,t)-f(v,x,t)\bigr]\phi_{n}{g_{n}}(u - v)\,dx\,dt. \end{aligned}$$

In the first place,

3.3 $$ \int_{\varOmega} a(x) \bigl( \vert \nabla u \vert ^{p - 2} \nabla u - \vert \nabla v \vert ^{p - 2}\nabla v \bigr) \cdot\nabla(u - v)g'{_{n}}(u-v)\phi_{n}(x)\,dx\geq0. $$

By Lemma 3.2, using the Lebesgue dominated convergence theorem,

3.4 $$\begin{aligned}& \lim_{n\rightarrow\infty} \int_{\tau}^{s} \int_{\varOmega}\phi _{n}(x){g_{n}}(u - v) \frac{\partial(u - v)}{\partial t}\,dx\,dt \\& \quad = \lim_{n\rightarrow\infty} \int_{\tau}^{s} \int_{\varOmega}\frac {\partial(\phi_{n}(x)G_{n}(u-v))}{\partial t}\,dx\,dt \\& \quad = \lim_{n\rightarrow\infty} \int_{\varOmega}\phi _{n}(x)\bigl[G_{n}(u-v) (x,s)-G_{n}(u-v) (x,\tau)\bigr]\,dx \\& \quad = \int_{\varOmega}|u-v|(x,s)\,dx- \int_{\varOmega}|u-v|(x,\tau)\,dx. \end{aligned}$$

Since $\nabla\phi_{n}=n\nabla a(x)$ when $x\in\varOmega\setminus\varOmega _{\frac{1}{n}}$, in the other places, it is identical to zero, by the assumption of (1.19), we have

3.5 $$\begin{aligned}& \biggl\vert \int_{\varOmega} a(x) \bigl( \vert \nabla u \vert ^{p- 2} \nabla u - \vert \nabla v \vert ^{p- 2}\nabla v \bigr) \cdot \nabla \phi_{n} g{_{n}}(u-v)\,dx \biggr\vert \\& \quad = \biggl\vert \int_{\varOmega\setminus\varOmega_{\frac{1}{n}}}a(x) \bigl( \vert \nabla u \vert ^{p- 2} \nabla u - \vert \nabla v \vert ^{p - 2}\nabla v \bigr) \cdot\nabla \phi_{n} g{_{n}}(u-v)\,dx \biggr\vert \\& \quad \leq n \int_{\varOmega\setminus\varOmega_{\frac{1}{n}}} a(x) \bigl( \vert \nabla u \vert ^{p - 1}+ \vert \nabla v \vert ^{p- 1} \bigr)|\nabla a g{_{n}}(u-v)| \,dx \\& \quad \leq c n \biggl( \int_{\varOmega\setminus\varOmega_{\frac{1}{n}}} a(x) \bigl( \vert \nabla u \vert ^{p}+ \vert \nabla v \vert ^{p} \bigr)\,dx \biggr)^{\frac{p-1}{p}} \biggl( \int_{\varOmega\setminus\varOmega_{\frac{1}{n}}} a(x) |\nabla a|^{p}\,dx \biggr)^{\frac{1}{p}} \\& \quad \leq c \biggl[ \biggl( \int_{\varOmega\setminus\varOmega_{\frac {1}{n}}}a(x)|\nabla u|^{p}\,dx \biggr)^{\frac{p-1}{p}}+ \biggl( \int _{\varOmega\setminus\varOmega_{\frac{1}{n}}}a(x)|\nabla v|^{p}\,dx \biggr)^{\frac{p-1}{p}} \biggr] \\& \qquad {}\cdot n \biggl( \int_{\varOmega\setminus\varOmega_{\frac{1}{n}}} a(x) |\nabla a|^{p}\,dx \biggr)^{\frac{1}{p}}. \end{aligned}$$

Since $a(x)\in C^{1}(\overline{\varOmega})$, by (3.5),

3.6 $$\begin{aligned}& \lim_{n\rightarrow\infty} \biggl\vert \int_{\tau}^{s} \int_{\varOmega} a(x) \bigl( \vert \nabla u \vert ^{p- 2} \nabla u - \vert \nabla v \vert ^{p- 2}\nabla v \bigr) \cdot\nabla \phi_{n} g_{n}(u-v)\,dx\,dt \biggr\vert \\& \quad \leq c\lim_{n\rightarrow\infty} \int_{\tau}^{s} \biggl( \int_{\varOmega \setminus\varOmega_{\frac{1}{n}}} a(x) |\nabla a|^{p}\,dx \biggr)^{\frac{1}{p}}\,dt \\& \quad =0. \end{aligned}$$

In the second place, since $b_{i}(s,x,t)$ satisfies the condition (1.16)

$$\bigl\vert b_{i}(u,x,t)-b_{i}(v,x,t) \bigr\vert \leq c g_{i}(x) \vert u-v \vert ,\qquad \int_{\varOmega } \bigl\vert g_{i}(x) \bigr\vert ^{\frac{p}{p-1}}a(x)^{-\frac{1}{p-1}}\,dx< \infty, $$

using the Lebesgue dominated convergence theorem, we have

3.7 $$\begin{aligned}& \lim_{n\rightarrow\infty} \biggl\vert \int_{\tau}^{s} \int_{{\varOmega }}\bigl[b_{i}(u,x,t)-b_{i}(v,x,t) \bigr](u-v)_{x_{i}}\phi_{n}{g'_{n}}(u - v)\,dx\,dt \biggr\vert \\& \quad \leq c \lim_{n\rightarrow\infty} \int_{\tau}^{s} \int_{{\varOmega \setminus\varOmega_{\frac{1}{n}}}} \bigl\vert {g'_{n}}(u - v) (u-v)g_{i}(x)a^{-\frac{1}{p}} \bigr\vert \bigl\vert a(x)^{\frac {1}{p}}(u-v)_{x_{i}}\phi_{n} \bigr\vert \,dx\,dt \\& \quad \leq c\lim_{n\rightarrow\infty} \biggl( \int_{\tau}^{s} \int _{\varOmega\setminus\varOmega_{\frac{1}{n}}}a(x) \bigl(|\nabla u|^{p}+|\nabla v|^{p} \bigr)\,dx\,dt \biggr)^{\frac{1}{p}} \\& \qquad {}\cdot \biggl( \int_{\tau}^{s} \int_{\varOmega} \bigl\vert {g'_{n}}(u - v) (u-v)g_{i}(x)a(x)^{-\frac{1}{p}} \bigr\vert ^{\frac{p}{p-1}}\,dx\,dt \biggr)^{\frac{p-1}{p}} \\& \quad =0. \end{aligned}$$

Last but not least, by condition (1.18) and $g_{i}(x)|_{x\in\partial \varOmega}=0$, we clearly have

3.8 $$\begin{aligned}& \lim_{n\rightarrow\infty} \biggl\vert \int_{\tau}^{s} \int_{{\varOmega }}\bigl[b_{i}(u,x,t)-b_{i}(v,x,t) \bigr]\phi_{nx_{i}}{g_{n}}(u - v)\,dx\,dt \biggr\vert \\& \quad \leq\lim_{n\rightarrow\infty} \int_{\tau}^{s}n \int_{{\varOmega }} \vert u-v \vert \bigl\vert g_{i}(x) \bigr\vert \vert a_{x_{i}} \vert \,dx\,dt \\& \quad \leq c\lim_{n\rightarrow\infty} \int_{\tau}^{s}n \int_{{\varOmega }} \bigl\vert g_{i}(x) \bigr\vert \vert a_{x_{i}} \vert \,dx\,dt \\& \quad = \int_{\tau}^{s} \int_{\partial\varOmega} \bigl\vert g_{i}(x) \bigr\vert \vert a_{x_{i}} \vert \,d\varSigma \,dt \\& \quad =0, \end{aligned}$$

3.9 $$\begin{aligned}& \lim_{n\rightarrow\infty} \biggl\vert \int_{\tau}^{s} \int_{{\varOmega }}\bigl[f(u,x,t)-f(v,x,t)\bigr](u-v) \phi_{n}{g_{n}}(u - v)\,dx\,dt \biggr\vert \\& \quad \leq \int_{\tau}^{s} \int_{\varOmega}|u-v|\,dx\,dt \\& \quad =0. \end{aligned}$$

Now, let $n\rightarrow\infty$ in (3.2). Then

3.10 $$\begin{aligned}& \int_{\varOmega}\bigl\vert u(x,s) - v(x,s) \bigr\vert \,dx \\& \quad \leqslant \int _{\varOmega}\bigl\vert u(x,\tau) - {v(x,\tau)} \bigr\vert \,dx +c \biggl( \int_{0}^{t} \int_{\varOmega}|u-v|\,dx\,dt \biggr)^{l},\quad \forall t \in[0,T), \end{aligned}$$

where $l\leq1$

By (3.10), we easily to get

$$\int_{\varOmega}\bigl\vert u(x,s) - v(x,s) \bigr\vert \,dx \leqslant \int _{\varOmega}\bigl\vert u(x,\tau) - {v(x,\tau)} \bigr\vert \,dx, $$

and by the arbitrariness of *τ*, we have

$$\int_{\varOmega}\bigl\vert u(x,s) - v(x,s) \bigr\vert \,dx \leqslant \int _{\varOmega}\bigl\vert {u_{0}}(x) - {v_{0}}(x) \bigr\vert \,dx. $$

 □

## Proofs of Theorem 1.6 and Theorem 1.7

### Proof of Theorem 1.6

Let $u(x,t)$ and $v(x,t)$ be two weak solutions of Eq. (1.4) with the initial values $u_{0}(x)$, $v_{0}(x)$, respectively.

For large enough *m*, let

4.1 $$ \phi_{m}(x)= \textstyle\begin{cases} 1, & \mbox{if } x\in\varOmega_{\frac{1}{m}}, \\ ma(x), & \mbox{if } x\in\varOmega\setminus\varOmega_{\frac{1}{m}}. \end{cases} $$

By a limit process, we can choose $\chi_{[\tau,s]}{g_{n}}(\phi_{m}(u - v))$ as the test function, then

4.2 $$\begin{aligned}& \int_{\tau}^{s} \int_{{\varOmega}} {g_{n}}\bigl(\phi_{m}(u - v) \bigr)\frac {\partial(u - v)}{\partial t}\,dx\,dt \\& \qquad {}+ \int_{\tau}^{s} \int_{{\varOmega}} a(x) \bigl( \vert \nabla u \vert ^{p - 2} \nabla u - \vert \nabla v \vert ^{p- 2}\nabla v \bigr) \cdot\nabla(u - v)g'_{n}\bigl(\phi_{m}(u - v)\bigr) \phi_{m}(x)\,dx\,dt \\& \qquad {}+ \int_{\tau}^{s} \int_{{\varOmega}} a(x) \bigl( \vert \nabla u \vert ^{p(x) - 2} \nabla u - \vert \nabla v \vert ^{p(x) - 2}\nabla v \bigr) \cdot(u - v)g'_{n}\bigl(\phi_{m}(u - v)\bigr)\nabla\phi _{n}\, dx\, dt \\& \qquad {}+ \int_{\tau}^{s} \int_{{\varOmega}}\bigl[b_{i}(u,x,t)-b_{i}(v,x,t) \bigr](u-v)\phi _{mx_{i}}{g'_{n}}\bigl( \phi_{m}(u - v)\bigr)\,dx\,dt \\& \qquad {}+ \int_{\tau}^{s} \int_{{\varOmega }}\bigl[b_{i}(u,x,t)-b_{i}(v,x,t) \bigr](u-v)_{x_{i}}\phi_{n}{g'_{n}} \bigl(\phi_{m}(u - v)\bigr)\,dx\,dt \\& \quad = \int_{\tau}^{s} \int_{\varOmega}\bigl[f(u,x,t)-f(v,x,t)\bigr]g_{n}\bigl( \phi_{m}(u-v)\bigr)\,dx\,dt. \end{aligned}$$

Certainly, we still have

4.3 $$ \int_{\varOmega} a(x) \bigl( \vert \nabla u \vert ^{p - 2} \nabla u - \vert \nabla v \vert ^{p - 2}\nabla v \bigr) \cdot\nabla(u - v)g'{_{n}}\bigl(\phi_{m}(u - v)\bigr) \phi_{m}(x)\,dx\geq0. $$

By Lemma 3.2, using the Lebesgue dominated convergence theorem,

4.4 $$\begin{aligned}& \lim_{m\rightarrow\infty}\lim_{n\rightarrow\infty} \int_{\tau }^{s} \int_{\varOmega}{g_{n}}\bigl(\phi_{m}(u - v) \bigr)\frac{\partial(u - v)}{\partial t}\,dx\,dt \\& \quad = \lim_{m\rightarrow\infty}\lim_{n\rightarrow\infty} \int_{\tau }^{s} \int_{\varOmega}\frac{\partial G_{n}(\phi_{m}(u - v))}{\partial t}\,dx\,dt \\& \quad = \lim_{m\rightarrow\infty}\lim_{n\rightarrow\infty} \int_{\varOmega}\frac{1}{\phi_{m}(x)}\bigl[G_{n}\bigl( \phi_{m}(u - v)\bigr) (x,s)-G_{n}\bigl(\phi_{m}(u - v)\bigr) (x,\tau)\bigr]\,dx \\& \quad = \int_{\varOmega}|u-v|(x,s)\,dx- \int_{\varOmega}|u-v|(x,\tau)\,dx. \end{aligned}$$

As before, $\nabla\phi_{m}=m\nabla a(x)$ when $x\in\varOmega\setminus \varOmega_{\frac{1}{m}}$, in the other places, it is identical to zero, by the fact that

$$\begin{aligned}& \lim_{n\rightarrow\infty}g'_{n}(s)s=0, \\& \int_{\varOmega}a(x)^{1-p}|\nabla a|^{p}\,dx< \infty, \end{aligned}$$

using the Lebesgue dominated convergence theorem, we have

4.5 $$\begin{aligned}& \lim_{n\rightarrow\infty} \biggl\vert \int_{\varOmega} a(x) \bigl( \vert \nabla u \vert ^{p- 2} \nabla u - \vert \nabla v \vert ^{p- 2}\nabla v \bigr) \nabla \phi_{m} (u-v)g'_{n}\bigl(\phi_{m}(u - v)\bigr)\,dx \biggr\vert \\& \quad =\lim_{n\rightarrow\infty} \biggl\vert \int_{\varOmega\setminus\varOmega _{\frac{1}{m}}}a(x) \bigl( \vert \nabla u \vert ^{p- 2} \nabla u - \vert \nabla v \vert ^{p - 2}\nabla v \bigr) \frac{\nabla\phi _{m}}{\phi_{m}}\phi_{m} (u-v)g'_{n}\bigl( \phi_{m}(u - v)\bigr)\,dx \biggr\vert \\& \quad \leq c\lim_{n\rightarrow\infty} \biggl( \int_{\varOmega\setminus \varOmega_{\frac{1}{m}}} a(x) \bigl( \vert \nabla u \vert ^{p}+ \vert \nabla v \vert ^{p} \bigr)\,dx \biggr)^{\frac{p-1}{p}} \\& \qquad {}\cdot \biggl( \int_{\varOmega\setminus\varOmega_{\frac{1}{m}}} a(x) \biggl\vert \frac{\nabla a}{a} \phi_{m} (u-v)g'_{n}\bigl(\phi_{m}(u - v)\bigr) \biggr\vert ^{p}\,dx \biggr)^{\frac{1}{p}} \\& \quad = 0. \end{aligned}$$

In the second place, since $b_{i}(s,x,t)$ satisfies the condition (1.18)

$$\bigl\vert b_{i}(u,x,t)-b_{i}(v,x,t) \bigr\vert \leq c g_{i}(x) \vert u-v \vert , \qquad \int_{\varOmega } \bigl\vert g_{i}(x) \bigr\vert ^{\frac{p}{p-1}}a(x)^{-\frac{1}{p-1}}\,dx< \infty, $$

using the Lebesgue dominated convergence theorem, we have

4.6 $$\begin{aligned}& \lim_{n\rightarrow\infty} \biggl\vert \int_{\tau}^{s} \int_{{\varOmega }}\bigl[b_{i}(u,x,t)-b_{i}(v,x,t) \bigr](u-v)_{x_{i}}\phi_{m}{g'_{n}} \bigl(\phi_{m}(u - v)\bigr)\,dx\,dt \biggr\vert \\& \quad \leq c \lim_{n\rightarrow\infty} \int_{\tau}^{s} \int_{{\varOmega }} \bigl\vert {g'_{m}}\bigl( \phi_{m}(u - v)\bigr)\phi _{m}(u-v)\bigl[b_{i}(u,x,t)-b_{i}(v,x,t) \bigr]a^{-\frac{1}{p}} \bigr\vert \\& \qquad {}\times\bigl\vert a^{\frac {1}{p}}(u-v)_{x_{i}} \bigr\vert \,dx\,dt \\& \quad \leq c\lim_{n\rightarrow\infty} \biggl( \int_{\tau}^{s} \int _{\varOmega}a\bigl(|\nabla u|^{p}+|\nabla v|^{p}\bigr)\,dx\,dt \biggr)^{\frac{1}{p}} \\& \qquad {}\times\biggl( \int_{\tau}^{s} \int_{\varOmega} \bigl\vert {g'_{n}}(u - v) (u-v)g_{i}(x)a^{-\frac{1}{p}} \bigr\vert ^{\frac{p}{p-1}}\,dx\,dt \biggr)^{\frac{p-1}{p}} \\& \quad =0. \end{aligned}$$

Last but not least, by

$$\int_{\varOmega} \biggl\vert \frac{a_{x_{i}}}{a} \biggr\vert \,dx \leq \biggl( \int _{\varOmega} \bigl\vert a(x)^{-\frac{1}{p}}g_{i}(x) \bigr\vert ^{\frac{p}{p-1}}\,dx \biggr)^{\frac{p-1}{p}} \biggl( \int_{\varOmega}a(x) \biggl\vert \frac{\nabla a}{a} \biggr\vert ^{p}\,dx \biggr)^{\frac{1}{p}}\leq c, $$

using the Lebesgue dominated convergence theorem, we have

4.7 $$\begin{aligned}& \lim_{n\rightarrow\infty} \biggl\vert \int_{\tau}^{s} \int_{{\varOmega }}\bigl[b_{i}(u,x,t)-b_{i}(v,x,t) \bigr](u-v)\phi_{mx_{i}}{g'_{n}}\bigl( \phi_{m}(u - v)\bigr)\,dx\,dt \biggr\vert \\& \quad \leq\lim_{n\rightarrow\infty} \int_{\tau}^{s} \int_{{\varOmega }} \bigl\vert \phi_{m}(u-v){g'_{n}} \bigl(\phi_{m}(u - v)\bigr) \bigr\vert \bigl\vert g_{i}(x) (u-v) \bigr\vert \biggl\vert \frac {a_{x_{i}}}{a} \biggr\vert \,dx\,dt \\& \quad =0, \end{aligned}$$

4.8 $$\begin{aligned}& \lim_{n\rightarrow\infty} \biggl\vert \int_{\tau}^{s} \int_{{\varOmega }}\bigl[f(u,x,t)-f(v,x,t)\bigr](u-v){g_{n}} \bigl(\phi_{m}(u - v)\bigr)\,dx\,dt \biggr\vert \\& \quad \leq \int_{\tau}^{s} \int_{\varOmega}|u-v|\,dx\,dt \\& \quad =0. \end{aligned}$$

Now, let $n\rightarrow\infty$ in (5.2). Then

$$\begin{aligned}& \int_{\varOmega}\bigl\vert u(x,s) - v(x,s) \bigr\vert \,dx \\& \quad \leqslant \int _{\varOmega}\bigl\vert u(x,\tau) - {v(x,\tau)} \bigr\vert \,dx +c \biggl( \int_{0}^{t} \int_{\varOmega}|u-v|\,dx\,dt \biggr)^{l},\quad \forall t \in[0,T), \end{aligned}$$

where $l\leq1$.

By (4.8), we easily get

$$\int_{\varOmega}\bigl\vert u(x,s) - v(x,s) \bigr\vert \,dx \leqslant \int _{\varOmega}\bigl\vert u(x,\tau) - {v(x,\tau)} \bigr\vert \,dx, $$

and by the arbitrariness of *τ*, we have

$$\int_{\varOmega}\bigl\vert u(x,s) - v(x,s) \bigr\vert \,dx \leqslant \int _{\varOmega}\bigl\vert {u_{0}}(x) - {v_{0}}(x) \bigr\vert \,dx. $$

 □

### Proof of Theorem 1.7

Let $u(x,t)$ and $v(x,t)$ be two weak solutions of Eq. (1.4) with the initial values $u_{0}(x)$, $v_{0}(x)$, respectively. Let

4.9 $$ \varphi_{m}(x)= \textstyle\begin{cases} 1, & \mbox{if } x\in\varOmega_{\frac{2}{m}}, \\ m(a(x)-\frac{1}{m}), & \mbox{if } x\in\varOmega_{\frac{1}{m}}\varOmega _{\frac{2}{m}}, \\ 0, & \mbox{if } x\in\varOmega\setminus\varOmega_{\frac{1}{m}}. \end{cases} $$

By a limit process, we can choose $\chi_{[\tau,s]}{g_{n}}(\varphi _{m}(u - v))$ as the test function, then

4.10 $$\begin{aligned}& \int_{\tau}^{s} \int_{{\varOmega}} {g_{n}}\bigl(\varphi_{m}(u - v)\bigr)\frac {\partial(u - v)}{\partial t}\,dx\,dt \\& \qquad {}+ \int_{\tau}^{s} \int_{{\varOmega}} a(x) \bigl( \vert \nabla u \vert ^{p - 2} \nabla u - \vert \nabla v \vert ^{p- 2}\nabla v \bigr) \cdot\nabla(u - v)g'_{n}\bigl(\varphi_{m}(u - v)\bigr) \varphi_{m}(x)\,dx\,dt \\& \qquad {}+ \int_{\tau}^{s} \int_{{\varOmega}} a(x) \bigl( \vert \nabla u \vert ^{p(x) - 2} \nabla u - \vert \nabla v \vert ^{p(x) - 2}\nabla v \bigr) \cdot(u - v)g'_{n}\bigl(\varphi_{m}(u - v)\bigr)\nabla \varphi _{n}\, dx\, dt \\& \qquad {}+ \int_{\tau}^{s} \int_{{\varOmega }}\bigl[b_{i}(u,x,t)-b_{i}(v,x,t) \bigr](u-v)\varphi_{mx_{i}}{g'_{n}}\bigl( \varphi_{m}(u - v)\bigr)\,dx\,dt \\& \qquad {}+ \int_{\tau}^{s} \int_{{\varOmega }}\bigl[b_{i}(u,x,t)-b_{i}(v,x,t) \bigr](u-v)_{x_{i}}\varphi_{m}{g'_{n}} \bigl(\varphi_{m}(u - v)\bigr)\,dx\,dt \\& \quad = \int_{\tau}^{s} \int_{{\varOmega}}\bigl[f(u,x,t)-f(v,x,t)\bigr]{g_{n}}\bigl( \varphi _{m}(u - v)\bigr)\,dx\,dt. \end{aligned}$$

Similarly, we have

4.11 $$ \int_{\varOmega} a(x) \bigl( \vert \nabla u \vert ^{p - 2} \nabla u - \vert \nabla v \vert ^{p - 2}\nabla v \bigr) \cdot\nabla(u - v)g'{_{n}}\bigl(\varphi_{m}(u - v)\bigr) \varphi_{m}(x)\,dx\geq0 $$

and

4.12 $$\begin{aligned}& \lim_{m\rightarrow\infty}\lim_{n\rightarrow\infty} \int_{\tau }^{s} \int_{\varOmega}{g_{n}}\bigl(\varphi_{m}(u - v) \bigr)\frac{\partial(u - v)}{\partial t}\,dx\,dt \\& \quad = \lim_{m\rightarrow\infty}\lim_{n\rightarrow\infty} \int_{\tau }^{s} \int_{\varOmega}\frac{\partial G_{n}(\varphi_{m}(u - v))}{\partial t}\,dx\,dt \\& \quad = \lim_{m\rightarrow\infty}\lim_{n\rightarrow\infty} \int_{\varOmega}\frac{1}{\varphi_{m}(x)}\bigl[G_{n}\bigl( \varphi_{m}(u - v)\bigr) (x,s)-G_{n}\bigl( \varphi_{m}(u - v)\bigr) (x,\tau)\bigr]\,dx \\& \quad = \int_{\varOmega}|u-v|(x,s)\,dx- \int_{\varOmega}|u-v|(x,\tau)\,dx. \end{aligned}$$

As before, $\nabla\phi_{m}=m\nabla a(x)$ when $x\in\varOmega\setminus \varOmega_{\frac{1}{m}}$, in the other places, it is identical to zero. Since $\int_{\varOmega}a(x)|\nabla u|^{p}\,dx<\infty$, by that $a(x)>0$ when $x\in\varOmega$, we have

4.13 $$ \int_{\varOmega_{\frac{1}{m}}\setminus \varOmega_{\frac{1}{m}}}|\nabla u|^{p}\,dx\leq \int_{\varOmega_{\frac{1}{m}}}|\nabla u|^{p}\,dx< c(m), $$

which yields

4.14 $$ \int_{\varOmega_{\frac{1}{m}}\setminus \varOmega_{\frac{2}{m}}}|\nabla u|^{p-1}\,dx\leq \int_{\varOmega_{\frac{1}{m}}}|\nabla u|^{p-1}\,dx< c(m). $$

By the fact that

$$\lim_{n\rightarrow\infty}g'_{n}(s)s=0, $$

and the assumption of (1.20), using the Lebesgue dominated convergence theorem, we have

4.15 $$\begin{aligned}& \lim_{n\rightarrow\infty} \biggl\vert \int_{\varOmega} a(x) \bigl( \vert \nabla u \vert ^{p- 2} \nabla u - \vert \nabla v \vert ^{p- 2}\nabla v \bigr) \nabla \varphi_{m} (u-v)g'_{n}\bigl( \varphi_{m}(u - v)\bigr)\,dx \biggr\vert \\& \quad =\lim_{n\rightarrow\infty} \biggl\vert \int_{\varOmega_{\frac {1}{m}}\setminus\varOmega_{\frac{2}{m}}}a(x) \bigl( \vert \nabla u \vert ^{p- 2} \nabla u - \vert \nabla v \vert ^{p - 2}\nabla v \bigr) \frac{\nabla\varphi_{m}}{\phi_{m}}\varphi_{m} (u-v)g'_{n}\bigl( \varphi_{m}(u - v)\bigr)\,dx \biggr\vert \\& \quad \leq\lim_{n\rightarrow\infty} \int_{\varOmega_{\frac{1}{m}}\setminus \varOmega_{\frac{2}{m}}} \bigl( \vert \nabla u \vert ^{p- 1}+ \vert \nabla v \vert ^{p - 1} \bigr) \frac{|\nabla a|}{a(x)-\frac {1}{m}}| \varphi_{m} (u-v)g'_{n}\bigl( \varphi_{m}(u - v)\bigr)\,dx \\& \quad = 0. \end{aligned}$$

Since $b_{i}(s,x,t)$ is a Lipschitz function, using Lebesgue dominated convergence theorem, we have

4.16 $$ \lim_{n\rightarrow\infty} \biggl\vert \int_{\tau}^{s} \int_{{\varOmega }}\bigl[b_{i}(u,x,t)-b_{i}(v,x,t) \bigr](u-v)_{x_{i}}\varphi_{m}{g'_{n}} \bigl(\varphi_{m}(u - v)\bigr)\,dx\,dt \biggr\vert =0, $$

obviously.

Last but not least, by (1.21) using the Lebesgue dominated convergence theorem, we have

4.17 $$\begin{aligned}& \lim_{n\rightarrow\infty} \biggl\vert \int_{{\varOmega_{\frac {1}{m}}\setminus\varOmega_{\frac {2}{m}}}}\bigl[b_{i}(u,x,t)-b_{i}(v,x,t) \bigr](u-v)\phi_{mx_{i}}{g'_{n}}\bigl( \phi_{m}(u - v)\bigr)\,dx\,dt \biggr\vert \\& \quad \leq\lim_{n\rightarrow\infty} \int_{{\varOmega}} \bigl\vert \phi _{m}(u-v){g'_{n}} \bigl(\phi_{m}(u - v)\bigr) \bigr\vert \bigl\vert (u-v) \bigr\vert \biggl\vert \frac{\nabla a}{a(x)-\frac{1}{m}} \biggr\vert \,dx\,dt \\& \quad =0, \end{aligned}$$

4.18 $$\begin{aligned}& \lim_{n\rightarrow\infty} \biggl\vert \int_{\tau}^{s} \int_{\varOmega }\bigl[f(u,x,t)-f(v,x,t)\bigr](u-v){g_{n}} \bigl(\varphi_{m}(u - v)\bigr)\,dx\,dt \biggr\vert \\& \quad \leq \int_{\tau}^{s} \int_{\varOmega}|u-v|\,dx\,dt. \end{aligned}$$

Now, after letting $n\rightarrow\infty$, let $m\rightarrow\infty$ in (4.10). Then we have the conclusion. □

## The usual boundary value condition

By Lemma 2.2, if $\int_{\varOmega}a(x)^{-\frac{1}{p-1}}\,dx<\infty$, then we can define the trace of *u* on the boundary *∂Ω*. If one imposes the usual boundary value condition (1.7), the stability of the weak solutions is true. For the completeness of the paper, we also give this conclusion and its proof here.

### Theorem 5.1

*Let* $p>1$, $\int_{\varOmega}a(x)^{-\frac {1}{p-1}}\,dx<\infty$, $f(s,x,t)$ *and* $b_{i}(s,x,t)$ *be Lipschitz functions*. *If* $u(x,t)$, $v(x,t)$ *are two solutions of Eq*. (1.4) *with the usual homogeneous value condition*,

5.1 $$ u(x,t)=v(x,t)=0,\quad (x,t)\in\partial\varOmega\times(0,T), $$

*and with the initial values* $u_{0}(x)$, $v_{0}(x)$, *respectively*, *then*

$$\int_{\varOmega} \bigl\vert u(x,t)-v(x,t) \bigr\vert \,dx\leq \int_{\varOmega} \bigl\vert u_{0}(x)-v_{0}(x) \bigr\vert \,dx. $$

### Proof

By a limit process, we can choose $\chi_{[\tau ,s]}{g_{n}}(u - v)$ as a test function. Then

5.2 $$\begin{aligned}& \int_{\tau}^{s} \int_{\varOmega} g_{n}(u - v)\frac{\partial(u - v)}{\partial t}\,dx\,dt \\& \qquad {}+ \int_{\tau}^{s} \int_{\varOmega} a(x) \bigl( \vert \nabla u \vert ^{p- 2} \nabla u - \vert \nabla v \vert ^{p- 2}\nabla v \bigr) \cdot \nabla(u - v)g'_{n}(u-v) \,dx\,dt \\& \qquad {}+ \int_{\tau}^{s} \int_{\varOmega}\bigl[b_{i}(u,x,t))-b_{i}(v,x,t) \bigr](u - v)_{x_{i}}g'_{n}(u-v) \,dx\,dt \\& \quad = \int_{\tau}^{s} \int_{\varOmega}\bigl[f(u,x,t)-f(v,x,t)\bigr]g_{n}(u-v)\,dx \,dt. \end{aligned}$$

As usual, we have

5.3 $$ \int_{\tau}^{s} \int_{\varOmega} a(x) \bigl( \vert \nabla u \vert ^{p- 2} \nabla u - \vert \nabla v \vert ^{p - 2}\nabla v \bigr) \cdot\nabla(u - v)g'_{n}(u-v)\,dx \,dt\geq0. $$

By Lemma 3.2,

5.4 $$\begin{aligned}& \lim_{n\rightarrow\infty} \int_{\tau}^{s} \int_{\varOmega}g_{n}(u - v)\frac{\partial(u - v)}{\partial t}\,dx\,dt \\& \quad =\lim_{n\rightarrow\infty} \int_{\varOmega }\bigl[G_{n}(u-v) (x,s)-G_{n}(u-v) (x,\tau)\bigr]\,dx \\& \quad = \int_{\varOmega}|u-v|(x,s)\,dx- \int_{\varOmega}|u-v|(x,\tau) \,dx. \end{aligned}$$

Moreover, similar to [15], we can prove that

5.5 $$\begin{aligned}& \lim_{n \to\infty} \int_{\varOmega} \bigl[b_{i}(u,x,t)- b_{i}(v,x,t) \bigr]{g_{n}}'(u - v) (u - v)_{x_{i}}\,dx = 0, \end{aligned}$$

5.6 $$\begin{aligned}& \lim_{n \to\infty} \biggl\vert \int_{\varOmega} \bigl[f(u,x,t)-f(v,x,t)\bigr]{g_{n}}(u - v)\,dx \biggr\vert \leq c \int_{\varOmega}|u-v|\,dx. \end{aligned}$$

Now, let $n\rightarrow\infty$ in (5.2). Then

$$\int_{\varOmega}{ \bigl\vert {u(x,s) - v(x,s)} \bigr\vert \, {d}x} \leqslant \int_{\varOmega}{ \bigl\vert {{u(x,\tau)} - {v(x,\tau)}} \bigr\vert \, {d}x} + \int_{\tau}^{s} \int_{\varOmega}|u(x,t)-v(x,t)|\,dx\,dt. $$

Let $\tau\rightarrow0$. Then, by the Gronwall inequality, we have

$$\int_{\varOmega}{ \bigl\vert {u(x,s) - v(x,s)} \bigr\vert \, {d}x} \leqslant \int_{\varOmega}{ \bigl\vert {{u_{0}(x)} - {v_{0}(x)}} \bigr\vert \, {d}x}. $$

Theorem 5.1 is proved. □

The interesting problem is that, since $a(x)$ may be degenerate on the boundary, the usual boundary value condition (1.7) is overdetermined [9]. Obviously, Theorem 1.8 has solved this problem partially.

### Proof of Theorem 1.8

If $u(x,t)$, $v(x,t)$ are two solutions of (1.4) with the initial values $u_{0}(x)$, $v_{0}(x)$, respectively, and with the same partial boundary value condition (1.21)

Let $\phi_{n}(x)$ be defined as in the proof of Theorem 1.5. By the assumption

$$u(x,t)=v(x,t)=0, \quad (x,t)\in\varSigma_{p}\times(0,T), $$

where

$$\varSigma_{p}=\varSigma_{1}=\bigl\{ x\in\partial\varOmega: a(x)a_{x_{i}}\neq0\bigr\} , $$

we can choose $\chi_{[\tau,s]}\phi_{n}(x){g_{n}}(u - v)$ as a test function. By the condition (1.23), similar to the proof Theorem 1.5, we are able to show the conclusion of Theorem 1.8, we omit the details here. □

## The uniqueness of the solution

In this section, we will prove Theorem 1.9.

### Proof of Theorem 1.9

Let $u(x,t)$, $v(x,t)$ be two solutions of Eq. (1.4) with the initial values $u_{0}(x)$, $v_{0}(x)$, respectively, and with

$$u(x,t)=v(x,t) =0,\quad (x,t)\in\varSigma_{p}\times(0,T), $$

where

$$\varSigma_{p}=\bigl\{ x\in\partial\varOmega: a(x)>0\bigr\} . $$

Then we may choose $\chi_{[\tau,s]}(u-v)a^{\beta}$ as a test function, where $\beta\geq1$ is a constant,

6.1 $$\begin{aligned}& \bigl\langle \bigl\langle (u-v)_{t}, \chi_{[\tau,s]}(u-v)a^{\beta} \bigr\rangle \bigr\rangle \\& \quad = \iint_{Q_{\tau s}}(u-v)a^{\beta}\frac{\partial(u-v)}{\partial t}\,dx\,dt \\& \quad =- \iint_{Q_{\tau s}}a(x) \bigl(|\nabla u|^{p-2}\nabla u-|\nabla v|^{p-2}\nabla v\bigr)\nabla\bigl[(u_{\varepsilon}-v_{\varepsilon})a^{\beta} \bigr] \,dx\,dt \\& \qquad {}-\sum_{i=1}^{N} \iint_{Q_{\tau s}}\bigl[b_{i}(u,x,t)-b_{i}(v,x,t) \bigr] \bigl[(u-v)a^{\beta}\bigr]_{x_{i}} \,dx\,dt \\& \qquad {}+ \iint_{Q_{\tau s}}a(x)^{\beta}\bigl[f(u,x,t)-f(v,x,t) \bigr](u-v)\,dx\,dt, \end{aligned}$$

where $Q_{\tau,s}=\varOmega\times(\tau,s)$. By that $|\nabla a|< c$, and

$$\iint_{Q_{T}}a(x) \vert \nabla u \vert ^{p}\, dx\, dt\leq c, \qquad \iint_{Q_{T}}a(x) \vert \nabla v \vert ^{p}\, dx\, dt\leq c, $$

by the Hölder inequality, we can show that

6.2 $$\begin{aligned}& \biggl\vert \iint_{Q_{\tau s}}(u-v)a(x) \bigl(|\nabla u|^{p-2}\nabla u-| \nabla v|^{p-2}\nabla v\bigr)\nabla a^{\beta}\,dx\,dt \biggr\vert \\& \quad \leq \iint_{Q_{\tau s}} \vert u-v \vert a(x) \bigl( \vert \nabla u \vert ^{p-1}+ \vert \nabla v \vert ^{p-1}\bigr) \bigl\vert \nabla a^{\beta} \bigr\vert \,dx\,dt \\& \quad \leq c \biggl( \int_{\tau}^{s} \int_{\varOmega}a^{1+p(\beta -1)} \vert u-v \vert ^{p} \,dx\,dt \biggr)^{\frac{1}{p}} \\& \quad \leq c \biggl( \int_{\tau}^{s} \int_{\varOmega}a^{\beta } \vert u-v \vert ^{2} \,dx\,dt \biggr)^{l}, \end{aligned}$$

where $l\geq1$. Here, we have the fact that

$$a^{1+p(\beta-1)}\leq c a^{\beta} $$

due to $\beta\geq1$.

As for the convection term,

6.3 $$\begin{aligned}& \iint_{Q_{\tau s}}\bigl[b_{i}(u,x,t)-b_{i}(v,x,t) \bigr] \bigl[(u-v)\xi_{\lambda }\bigr]_{x_{i}} \,dx\,dt \\& \quad = \iint_{Q_{\tau s}}\bigl[b_{i}(u,x,t)-b_{i}(v,x,t) \bigr](u-v)a^{\beta}_{ x_{i}} \,dx\,dt \\& \qquad {}+ \iint_{Q_{s}}\bigl[b_{i}(u,x,t)-b_{i}(v,x,t) \bigr](u-v)_{x_{i}}a^{\beta} \,dx\,dt. \end{aligned}$$

Since $\beta\geq2$, $|a_{x_{i}}|\leq|\nabla a|\leq c$, by the Hölder inequality, we have

6.4 $$\begin{aligned}& \iint_{Q_{\tau s}}\bigl[b_{i}(u,x,t)-b_{i}(v,x,t) \bigr](u-v)a^{\beta}_{x_{i}} \,dx\,dt \\& \quad = \int_{\tau}^{s} \int_{\varOmega_{\lambda }}\bigl[b_{i}(u,x,t)-b_{i}(v,x,t) \bigr](u-v)a^{\beta-1}|a_{x_{i}}|\,dx \\& \quad \leq \int_{\tau}^{s} \int_{\varOmega}|u-v|a^{\beta-1}\,dx \\& \quad \leq c \biggl( \int_{\tau}^{s} \int_{\varOmega}a^{\beta }|u-v|^{2}\,dx\,dt \biggr)^{\frac{1}{2}} \end{aligned}$$

and

6.5 $$\begin{aligned}& \biggl\vert \iint_{Q_{\tau s}}\bigl[b_{i}(u,x,t)-b_{i}(v,x,t) \bigr](u-v)_{x_{i}}a^{\beta} \,dx\,dt \biggr\vert \\& \quad \leq\sum_{i=1}^{N} \biggl( \int_{\tau}^{s} \int_{\varOmega}a^{(\beta -\frac{1}{p})p'} \bigl\vert b_{i}(u,x,t)-b_{i}(v,x,t) \bigr\vert ^{p'}\,dx\,dt \biggr)^{\frac{1}{p'}} \\& \qquad {}\times\biggl( \int_{\tau}^{s} \int_{\varOmega}a\bigl(|\nabla u|^{p}+|\nabla v|^{p}\bigr) \,dx\,dt \biggr)^{\frac{1}{p}} \\& \quad \leq c\sum_{i=1}^{N} \biggl( \int_{\tau}^{s} \int_{\varOmega}a{\biggl(\beta -\frac{1}{p} \biggr)p'}\bigl\vert b_{i}(u,x,t)-b_{i}(v,x,t) \bigr\vert \bigr)^{p'}\,dx\,dt \biggr)^{\frac{1}{p'}} \\& \quad \leq c \biggl( \int_{\tau}^{s} \int_{\varOmega}a^{\beta }|u-v|^{p'}\,dx\,dt \biggr)^{\frac{1}{p'}} \\& \quad \leq c \biggl( \int_{\tau}^{s} \int_{\varOmega}a^{\beta }|u-v|^{2}\,dx\,dt \biggr)^{l}. \end{aligned}$$

Since $f(s,x,t)$ is a continuous function, $\|u\|_{L^{\infty}(Q_{T})}\leq c$, $\|u\|_{L^{\infty}(Q_{T})}\leq c$,

6.6 $$ \biggl\vert \iint_{Q_{\tau s}}a(x)^{\beta }\bigl[f(u,x,t)-f(v,x,t) \bigr](u-v)\,dx\,dt \biggr\vert \leq c \iint_{Q_{\tau s}}a(x)^{\beta}|u-v|\,dx\,dt , $$

obviously.

By Lemma 3.1,

6.7 $$\begin{aligned}& \iint_{Q_{\tau s}}(u-v)a^{\beta}\frac{\partial(u-v)}{\partial t}\,dx\,dt \\& \quad = \iint_{Q_{\tau s}}(u-v)\sqrt{a^{\beta}}\frac{\sqrt{a^{\beta }}\partial(u-v)}{\partial t} \\& \quad = \int_{\varOmega}a^{\beta}\bigl[u(x,s)-v(x,s) \bigr]^{2}\,dx- \int_{\varOmega}a^{\beta }\bigl[u(x,\tau)-v(x,\tau) \bigr]^{2}\,dx. \end{aligned}$$

From (6.2)–(6.7), by (6.1), we have

6.8 $$\begin{aligned}& \int_{\varOmega}a^{\beta}\bigl[u(x,s)-v(x,s) \bigr]^{2}\,dx- \int_{\varOmega}a^{\beta }\bigl[u(x,\tau)-v(x,\tau) \bigr]^{2}\,dx \\& \quad \leq c \biggl( \int_{\tau}^{s} \int_{\varOmega }a^{\beta} \bigl\vert u(x,t)-v(x,t) \bigr\vert ^{2}\,dx\,dt \biggr)^{l}, \end{aligned}$$

where $l\leq1$. By (6.8), we easily get

$$\int_{\varOmega}a^{\beta} \bigl\vert u(x,s) - v(x,s) \bigr\vert ^{2}\,dx \leqslant \int_{\varOmega}a^{\beta} \bigl\vert u(x,\tau) - {v(x, \tau)} \bigr\vert ^{2}\,dx, $$

and by the arbitrariness of *τ*, we have

$$\int_{\varOmega}a^{\beta} \bigl\vert u(x,s) - v(x,s) \bigr\vert ^{2}\,dx \leqslant \int_{\varOmega}a^{\beta} \bigl\vert {u_{0}}(x) - {v_{0}}(x) \bigr\vert ^{2}\,dx. $$

 □

## Conclusion

Compared with our previous work [10], not only is the equation considered in this paper more general, but also the conclusions are much better. In particular, the uniqueness of the weak solution based on a partial boundary value condition (Theorem 1.9) is always true. This conclusion is more or less beyond one’s imagination. Benedikt et al. had considered the equation

7.1 $$ {u_{t}} = \operatorname{div} \bigl({ \vert {\nabla u} \vert ^{p - 2}}\nabla u\bigr) + q(x)|u|^{\gamma-1}u , $$

and showed that the uniqueness of the solution is not true [16]. But Theorem 1.9 in this paper implies that, only if $u_{t}\in W'(Q_{T})$, the uniqueness of the solution to Eq. (7.1) is true.
